# A Portable Smartphone-Based Sensing System Using a 3D-Printed Chip for On-Site Biochemical Assays

**DOI:** 10.3390/s18114002

**Published:** 2018-11-16

**Authors:** Feiyi Wu, Min Wang

**Affiliations:** Institute of Microanalytical Systems, Department of Chemistry, Zhejiang University, Hangzhou 310058, China; wfy@zju.edu.cn

**Keywords:** smartphone, sensing system, chromogenic, signal capture, signal processing, organophosphorus pesticides, multi-assay, fruit juice

## Abstract

Recently, smartphone-based chromogenic sensing with paper-based microfluidic technology has played an increasingly important role in biochemical assays. However, generally there were three defects: (i) the paper-based chips still required complicated fabrication, and the hydrophobic boundaries on the chips were not clear enough; (ii) the chromogenic signals could not be steadily captured; (iii) the smartphone apps were restricted to the detection of specific target analytes and could not be extended for different assays unless reprogrammed. To solve these problems, in this study, a portable smartphone-based sensing system with a 3D-printed chip was developed. A 3D-printed imaging platform was designed to significantly reduce sensing errors generated during signal capture, and a brand-new strategy for signal processing in downloadable apps was established. As a proof-of-concept, the system was applied for detection of organophosphorus pesticides and multi-assay of fruit juice, showing excellent sensing performance. For different target analytes, the most efficient color channel could be selected for signal analysis, and the calibration equation could be directly set in user interface rather than programming environment, thus the developed system could be flexibly extended for other biochemical assays. Consequently, this study provides a novel methodology for smartphone-based biochemical sensing.

## 1. Introduction

In the past several years, biochemical assays with point-of-care devices have attracted enormous attentions because they provide great convenience in the fields of medicine diagnosis, food safety, drug detection, environment monitoring, and forensic analysis. The ubiquitous application of smartphones among almost people of all ages and income levels offers an opportunity for the development of mobile-detection systems. Recently, smartphone-based sensing systems have played increasingly important roles in biochemical assays [1].

Among the various sensing signals, chromogenic signals have been broadly utilized for smartphone-based sensing systems [2,3,4,5,6,7,8]. In contrast with a scanner, a smartphone is lightweight and portable, and it does not directly contact with the reagent zones. However, chromogenic signals are easily affected by the illumination conditions and position of the smartphone [9]. Some researchers have tried to reduce bias by using self-calibration approaches. For example, in a recent report on determination of urinary albumin by Mathaweesansurn et al. [3], the sample and a standard colorimetric strip were simultaneously captured in a single shot by a smartphone, and the printed reference colors on the standard colorimetric strip could represent the standard solutions to establish the calibration equation for every measurement. In addition, an Android app termed “Albumin smart test” was designed, and three frames displayed in the user interface of the app were employed for alignment between the test paper and the smartphone. Nevertheless, in our study, it was discovered that the illumination in a single shot was still not uniform (refer to Section 3.2.3).

To solve the imaging problem at the source, several groups have introduced auxiliary optical devices for smartphones [4,5,10]. For example, Wang et al. put the test paper on the top of a light-emitting diode (LED) lamp to prevent influence from ambient light [4]. Li et al. developed a 3D-printed attachment to increase measurement accuracy, in which 16 white LEDs were installed at the edges of a light guide plate placed between a reflector and a diffuser [5]. Oncescu et al. reported a smartphone case which had a slot for inserted test strips. The smartphone case was 3D-printed with opaque black material to isolate the test strips from external light, and a flash diffuser composed of polydimethylsiloxane (PDMS) membrane could diffuse light from the smartphone flash for uniform illumination [10].

On the other hand, microfluidic paper-based analytical devices (μPADs) with various designs were widely used in chromogenic detection [11], among which the flower-shaped design was a classic and representative type. The methods of wax printing [12], polystyrene flexographic printing [13], ink plotting [14,15], contact stamping [16], and plasma treatment [17] have been used to form the flower-shaped hydrophobic barriers of μPADs. Nevertheless, it is quite difficult to obtain hydrophobic barriers which could penetrate the entire paper thickness without penetrating into the expected hydrophilic channels. He et al. fabricated a μPAD via hydrophobization by coupling octadecyltrichlorosilane onto filter paper, followed by UV-lithography of the octadecyltrichlorosilane coating through a quartz mask which had a flower-shaped transparent pattern, thus the UV-exposed flower-shaped region turned hydrophilic and the masked region remained hydrophobic [18]. A similar method was reported by Asano et al. [19], and the difference between these two methods was that in the latter the photomask was fabricated by 3D-printing easily and rapidly. The defect of these two methods was that the paper would turn yellow and fragile after being UV-exposed. In a recent inspirational report by He et al. [20], a microfluidic analytical device was fabricated by adding cellulose suspension into the flower-shaped hollow channel network on a 3D-printed chip, and it was applied for assay of nitrite concentration. However, water evaporation and powder shrinkage might cause cracks in the filling areas, which would lead to interrupt of the flow in the channels.

In this study, a chip with a flower-shaped design was fabricated by 3D-printing and filled with cellulose powder instead of cellulose suspension. Consequently, the cracks caused by water evaporation and powder shrinkage in the filling areas were efficiently alleviated. In order to radically resolve the troublesome problem on signal capture, a 3D-printed imaging platform was originally developed for the chip with flower-shaped design. In virtue of the imaging platform, the chromogenic signals on the chip could be steadily and sensitively captured by a smartphone.

In the previous reports relevant to smartphone-based chromogenic detection, the smartphone apps were usually restricted to detection of several specific target analytes and could not be extended for detection of other analytes unless reprogrammed. In this study, a brand-new strategy for signal processing in downloadable smartphone apps was established. For different target analytes, with this strategy, the most efficient color channel could be selected for signal analysis, and the calibration equation could be directly set in user interface rather than programming environment, thus the developed system could be flexibly extended for different biochemical assays.

As a proof-of-concept, the developed system was applied for detection of organophosphorus pesticides (OPs) and on-site multi-assay of phenols, flavonoids, antioxidant capacity, and tyrosinase inhibition effect in fruit juice. More importantly, it provides a novel methodology for smartphone-based biochemical sensing.

## 2. Materials and Methods

### 2.1. Reagents

Indoxyl acetate, malathion, and aluminum chloride were purchased from Aladdin (Shanghai, China). Acetylcholinesterase (AChE), cellulose powder, gallic acid, rutin, l-ascorbic acid, cerium oxide nanoparticles, resveratrol, and l-3,4-dihydroxyphenylalanine were purchased from Macklin (Shanghai, China). Tyrosinase was purchased from Sangon (Shanghai, China). Fruit juice was purchased from a local market. AChE was dissolved in phosphate buffered saline (pH 7.4, 50 mg/mL). Tyrosinase was dissolved in phosphate buffered saline (pH 6.5, 0.5 mg/mL).

### 2.2. Fabrication of the Sensing System

**Figure 1:**
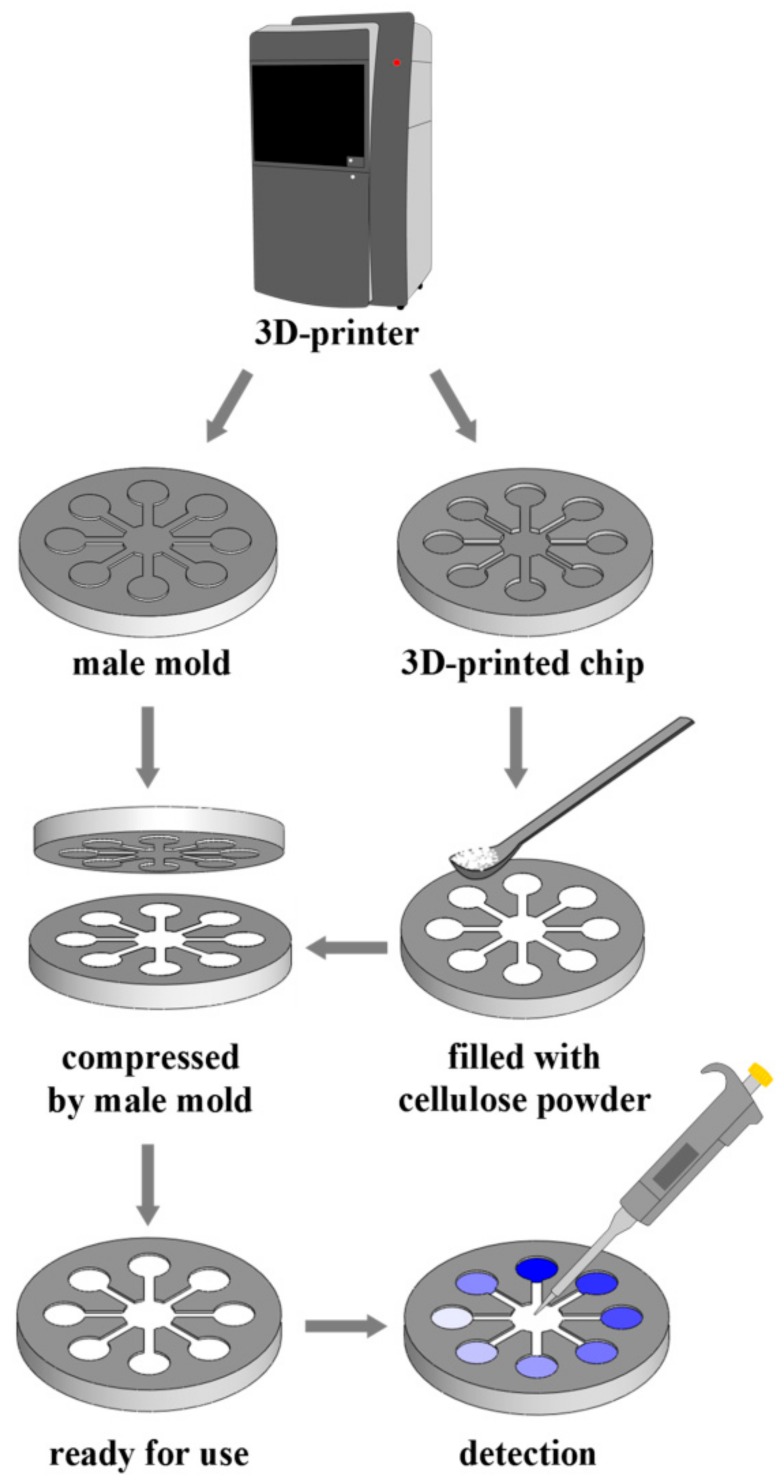
The fabrication process of the chip.

The 3D model of the chip with diameter of 50 mm and thickness of 5 mm was designed using a computer aided design (CAD) program, SolidWorks (Dassault Systemes, Paris, France). A flower-shaped hollow channel network was designed at the center of the chip, including a central zone with diameter of 10 mm, eight straight channels with width of 2 mm, and eight detection zones with diameter of 8 mm. The chip and a male mold with raised flower-shaped pattern were fabricated by a 3D-printer (3D Systems, Rock Hill, SC, USA) with light-sensitive resin as the printing material. Then, the flower-shaped hollow channel network of the chip was filled with cellulose powder (0.24 g), and the male mold was used to compress the cellulose powder into a flat layer. The schematic fabrication process of the chip is shown in Figure 1.

**Figure 2:**
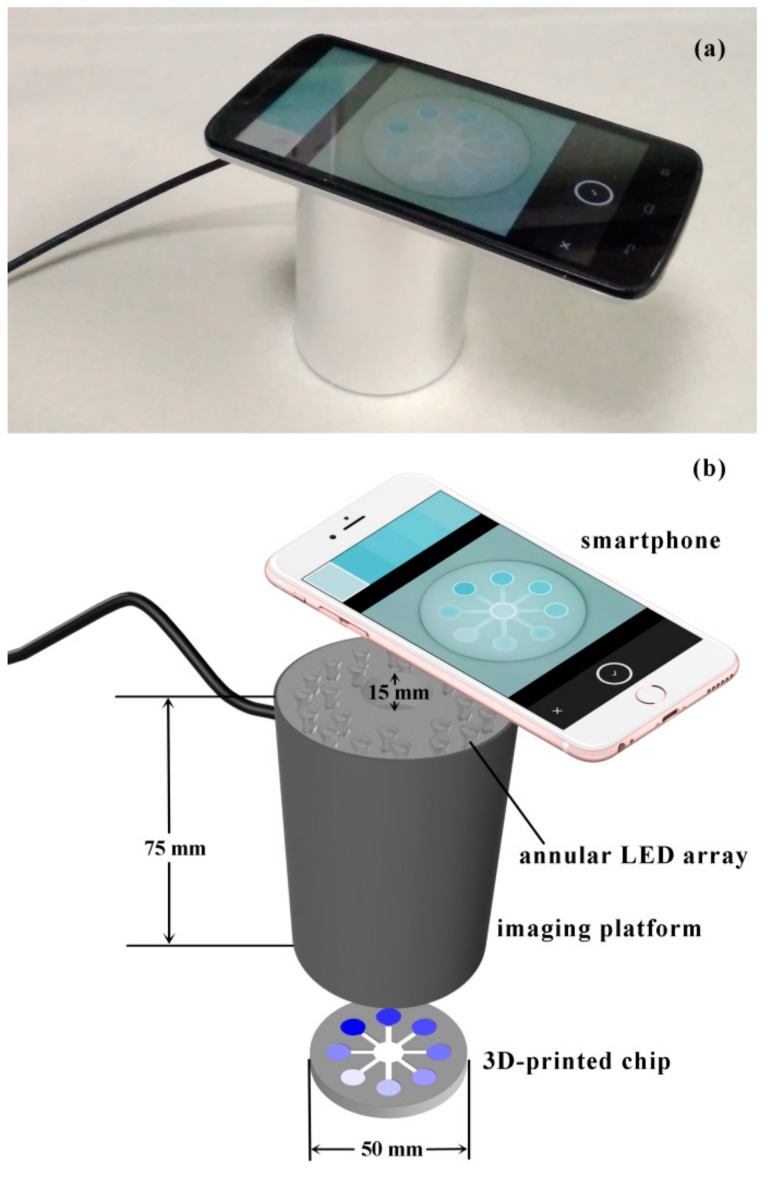
The image (**a**) and schematic diagram (**b**) of the developed system. In order to show the annular LED array, the opaque top surface of the imaging platform is drawn transparent in (**b**).

In order to capture steady and sensitive signals, an imaging platform was developed for the chip with flower-shaped design (Figure 2). The 3D model of the imaging platform was designed using SolidWorks. A window for a smartphone lens was designed at the top of the imaging platform (Figure 2b), while a “mouth” with diameter of 50 mm for the 3D-printed chip was designed at the bottom. Next, the imaging platform was fabricated by a 3D-printer (FARSOON, Changsha, China) with nylon as the printing material. On the ceiling of the imaging platform, 24 LEDs (eight inner and 16 outer ones) were installed facing the bottom (Figure 2b). In each round, LEDs were annularly arrayed with uniform interval. Finally, the outside of the imaging platform was wrapped with aluminum foil paper to further reduce light transmittance (Figure 2a).

### 2.3. Detection of OPs

**Figure 3:**
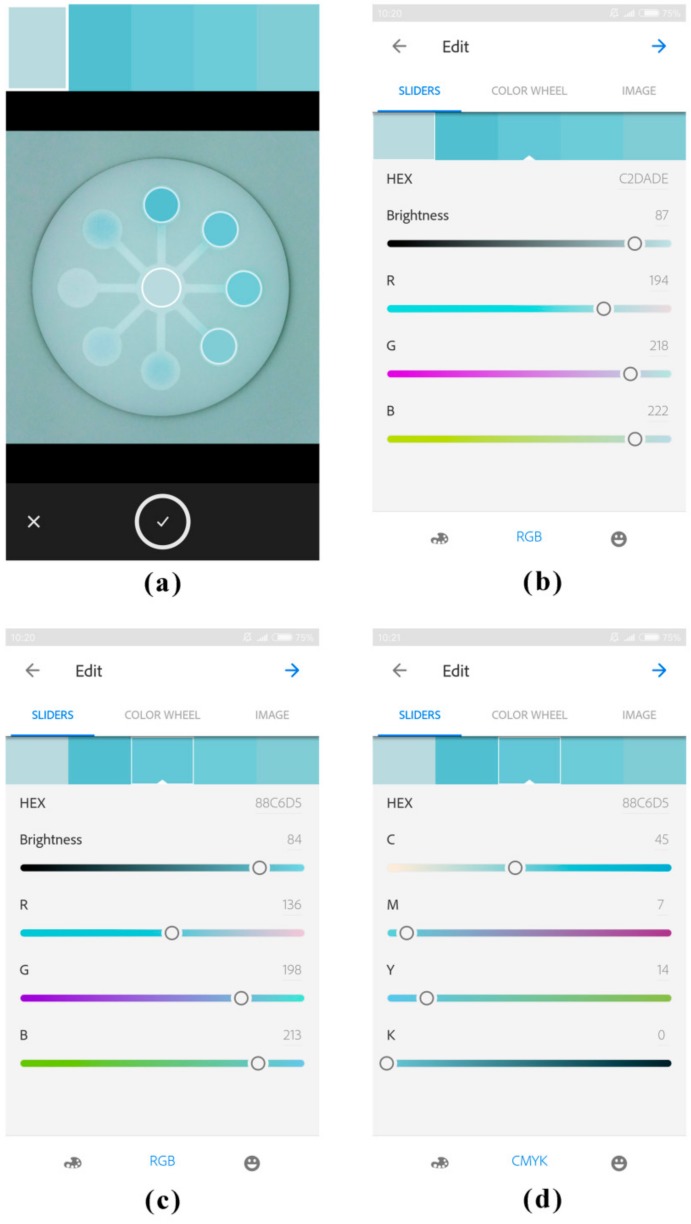
The established mode for chromogenic detection in Adobe Capture CC on the smartphone. (**a**) The representative color in each circle was displayed as a color card on the top of user interface. (**b**) The color information in R, G, and B channels of the first color card (background). (**c**) The color information in R, G, and B channels of the third color card (the second detection zone). (**d**) The color information in C, M, Y, and K channels of the third color card (the second detection zone).

First, 15 μL portions of AChE were individually pipetted into the eight detection zones on the chip. Next, 15 μL portions of malathion standard solutions or the sample solution were individually pipetted into the eight detection zones, followed by a 15 min inhibition time. Then, 180 μL of the colorless indoxyl acetate solution (8 mM) was pipetted into the central zone, and it would flow towards the eight detection zones by capillary force. Next, the chromogenic chip was inserted into the “mouth” of the imaging platform. After color development for 20 min, the chromogenic signals on the chip were captured by a smartphone with a downloadable app, Adobe Capture CC. As shown in Figure 3a, there were five circles which served as color pickers in Adobe Capture CC, and they could be moved on the smartphone touch-screen by fingers. The representative color in each circle was displayed as a color card on the top of user interface. The first circle could be moved onto the central zone of the chip representing the color intensity of background, and the other four could be moved onto four detection zones individually. As a result, signals from four detection zones of the chip could be analyzed simultaneously, thus signals from the total eight detection zones could be analyzed in two batches within a few minutes. The color information in R, G, B, C, M, Y, and K channels of the selected color card (highlighted by a white rectangular frame) would be displayed in the user interface (Figure 3b–d). The color intensities of the detection zones with background subtraction were obtained as the signal values.

**Figure 4:**
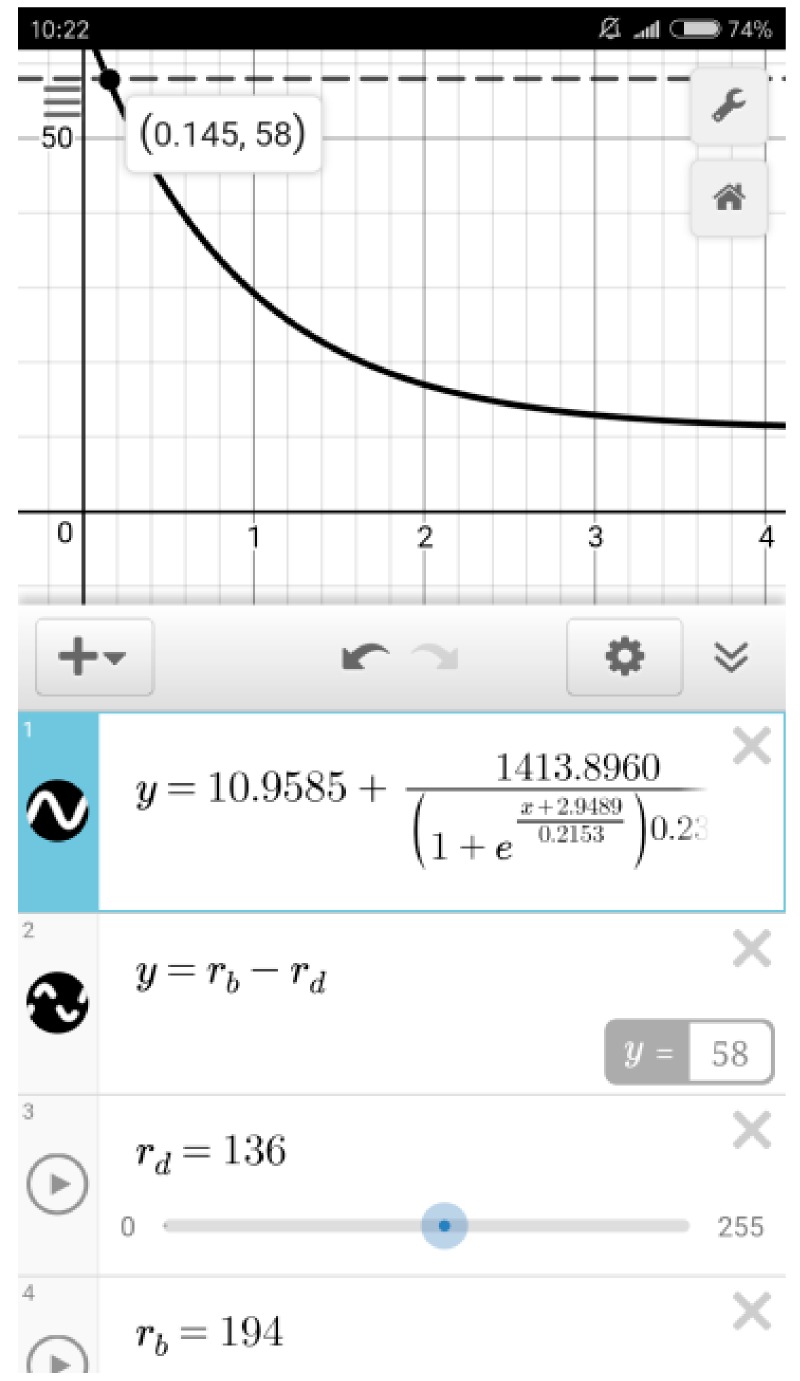
The established mode for malathion quantitation in Desmos on the smartphone.

The calibration equation for R value regression was input into another downloadable app, Desmos (Figure 4). Meanwhile, the function “*y* = *r_b_* − *r_d_*” was also input, where *r_b_* and *r_d_* represent the original R intensity of background and the detection zone respectively. Both *r_b_* and *r_d_* range from 0 to 255, and they could be set by simply moving the sliders on the smartphone touch-screen. Hence, the calculation result of the function “*y* = *r_b_* − *r_d_*” was the R value with background subtraction, and it would be automatically displayed at the lower right corner in the second column. In the plot area, the solid curve was the regression curve, and the dashed line was the R value of the detection zone (with background subtraction). The abscissa value of the intersection point of the regression curve and the dashed line was the detected malathion concentration (μg/mL), and it could be immediately calculated and displayed on the smartphone. Herefrom, a quantitative mode for malathion in Desmos had been established on the smartphone, and it could be saved in the accounts of users and directly invoked during detection.

### 2.4. Multi-Assay of Fruit Juice

Phenols and flavonoids in fruit juice contribute to multiple health-promoting benefits as they decrease the risks of cancer, coronary cardiopathy and neurodegenerative disease [21]. Antioxidants including vitamins, phenols, and flavonoids are able to reduce oxidative stress, created by reactive oxygen and nitrogen species (ROS and RNS), thus they can prevent aging and a wide variety of diseases [22]. Consequently, a need has arisen for fast and inexpensive detection of phenols, flavonoids, and total antioxidants in fruit juice. Tyrosinase plays a critical role in melanin production; however, excess production of melanin causes hyperpigmentation dysfunctions [22], which might become an aesthetic problem and lead to considerable psychological consequences to affected patients. On this basis, analysis of tyrosine inhibition effect in fruit juice would be of great value. Hence, in this study, the developed system was applied for on-site multi-assay of phenols, flavonoids, antioxidant capacity, and tyrosinase inhibition effect in fruit juice. Herein, *Rosa roxburghii* juice was multi-evaluated in an envisaged application of nutritional comparison on different sorts of fruit juice.

**Figure 5:**
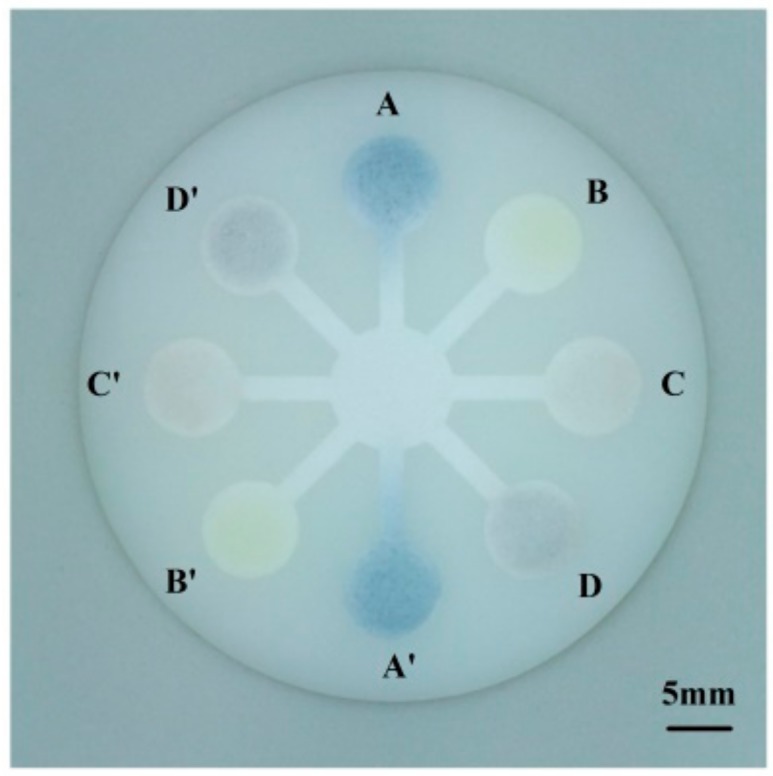
Multi-assay of phenols (A), flavonoids (B), antioxidant capacity (C), and tyrosinase inhibition effect (D) in fruit juice. (For interpretation of the references to color in this figure legend, please refer to the web version of the article.).

As shown in Figure 5, for assay of phenolic content, 15 μL portions of Folin-Ciocalteu reagent were individually pipetted into the detection zones A and A’. For assay of flavonoid content, 15 μL portions of a mixture of 0.09 M aluminum chloride solution and 0.12 M calcium acetate solution (1:1) were individually pipetted into the detection zones B and B’. For assay of antioxidant capacity, 15 μL portions of 0.058 M cerium oxide nanoparticles solution were individually pipetted into the detection zones C and C’. For assay of tyrosine inhibition effect, 15 μL portions of tyrosinase were individually pipetted into the detection zones D and D’.

*Rosa roxburghii* juice was filtered through 0.22 μm filter and diluted 10 times in distilled water. During detection, 180 μL of juice sample was pipetted into the central zone of the chip, and it would flow towards the eight detection zones by capillary force. After 5 min, 10 μL portions of 1 M sodium carbonate solution were individually pipetted into the detection zones A and A′, and 10 μL portions of 0.015 M l-3,4-dihydroxyphenylalanine were individually pipetted into the detection zones D and D′ (Figure 5). After subsequent 2 min, the chromogenic signals on the chip were captured by the smartphone via the imaging platform

**Figure 6:**
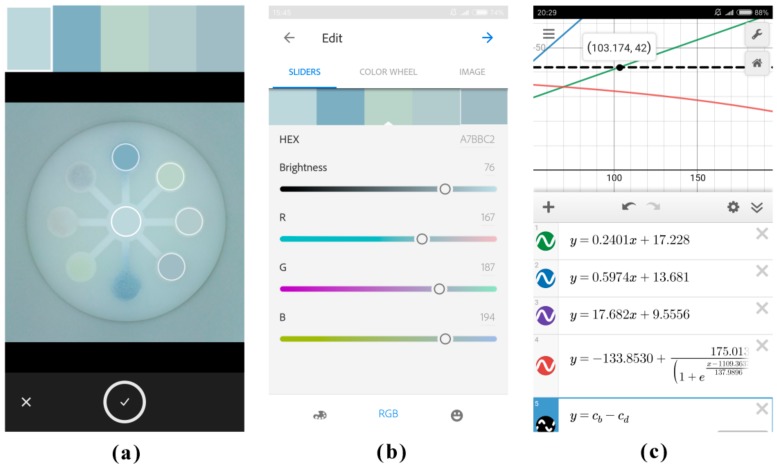
The established mode for multi-assay of fruit juice in Adobe Capture CC (**a**,**b**) and Desmos (**c**) on the smartphone. (For interpretation of the references to color in this figure legend, please refer to the web version of the article.).

In Adobe Capture CC, the first circle was moved onto the central zone of the chip representing the color intensity of background (Figure 6a), and the other four were individually moved onto detection zones A, B, C, and D. Therefore, signals for phenolic content, flavonoid content, antioxidant capacity, and tyrosine inhibition effect were analyzed simultaneously (Figure 6a,b). 

In Desmos, a pre-established quantitative mode for multi-assay of fruit juice was invoked (Figure 6c). The calibration equations for phenolic content, flavonoid content, antioxidant capacity, and tyrosine inhibition effect were individually displayed in the first, second, third, and fourth columns, while the regression curves of those were individually displayed as red, blue, purple, and green curves. The phenolic content, flavonoid content, antioxidant capacity, and tyrosine inhibition effect were individually expressed as μg gallic acid equivalent/mL, μg rutin equivalent/mL, mg L-ascorbic acid equivalent/mL, and μg resveratrol equivalent/mL. The function “*y* = *c_b_* − *c_d_*” was in the fifth column, where *c_b_* and *c_d_* represent the original color intensity of background and the detection zone respectively. Both *c_b_* and *c_d_* range from 0 to 255, and they could be set by simply moving the sliders on the smartphone touch-screen. The signal value of the detection zone (with background subtraction) would be displayed as a dashed black line in the plot area. Hence, the abscissa value of the intersection point of the dashed line and each solid regression curve was the detected level for each index (Figure 6c). For other sorts of fruit juice, the sensing signals for phenolic content, flavonoid content, antioxidant capacity, and tyrosine inhibition effect could also be simultaneously captured and processed on the smartphone, thus the nutrient contents and bioactivity function of different sorts of fruit juice could be on-site evaluated and compared.

## 3. Results and Discussion

### 3.1. Construction of the Sensing System

**Figure 7:**
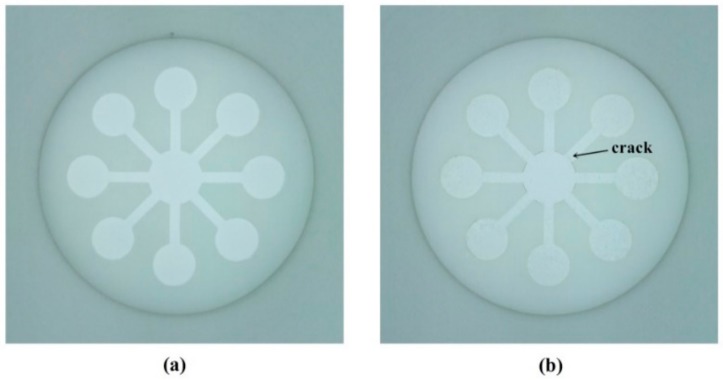
The chips filled with cellulose powder (**a**) and cellulose suspension (**b**) after 24 h.

Cellulose is a polysaccharide composed of β linked d-glucose units with the formula (C_6_H_10_O_5_)_n_, and it is able to generate flow by spontaneous capillary force. Therefore, the 3D-printed chip filled with cellulose powder was self-driven without the need for external power supplies. The hydrophilic zone was well-defined on the fabricated chip with clear and thorough boundary. Because the chip was filled with cellulose powder instead of cellulose suspension, the cracks caused by water evaporation and powder shrinkage in the filling areas were efficiently alleviated (shown in Figure 7). Besides, since the chip was made of light-sensitive resin rather than thermoplastic polymers (such as nylon), it could be printed with higher resolution.

During imaging, the chromogenic chip was inserted into the “mouth” of the imaging platform, and the smartphone was placed on the top of the imaging platform (Figure 2). The window on the imaging platform was a bit bigger than the smartphone lens, and it could be sheltered by the smartphone, thus the imaging platform could be integrated as a small attachment to the smartphone camera. The chromogenic chip, the imaging platform, and the smartphone together formed a chamber segregated with ambient stray light. Meanwhile, the smartphone was kept in a parallel position with the chromogenic chip, which could avoid distortion in the shapes of the detection zones caused by tilt. On the ceiling of the imaging platform, three LEDs (one inner and two outer) constituted a lighting unit in concordance with a detection zone on the chip. Consequently, eight lighting units were exactly corresponding with eight detection zones. The imaging platform was made of white nylon, thus its inner wall had an opaque white appearance with plenty of surface particles (see Figure S1 in ESI). As a result, the diffuse reflection of the inner wall was enhanced, and the lighting condition in the chamber was further homogenized. Hence, the lighting conditions on each detection zone were equal to one another, and the lighting conditions in each measurement were constant.

### 3.2. Detection of OPs

#### 3.2.1. Detection Principle

OPs have been widely used in agriculture to protect crops, vegetables, and fruits against insect invasion. However, they are acutely toxic to human through irreversible inactivation of AChE, which played an essential role in the hydrolysis of the neurotransmitter acetylcholine (ACh) [23]. Regarding to the concerns of food safety and environment protection, detection of OPs is highly demanded. Traditional methods for quantitative determination of OPs focus on high performance liquid chromatography (HPLC) or gas chromatography (GC) coupled with different detectors [24,25]. Although these methods can give sensitive and reliable results, most of them usually require time-consuming pretreatment, sophisticated equipment, and skilled manpower. To ameliorate these defects, some rapid detection approaches such as chromogenic approach [26] or electrochemical approach [27] have been developed. In this study, detection of OPs was performed by a chromogenic approach, and the chromogenic reaction is shown in Figure S2 (see in ESI). The colorless substrate (indoxyl acetate) was catalytically hydrolyzed into colorless indoxyl in the presence of AChE, and indoxyl was spontaneously oxidized into a blue product. Therefore, if the AChE activity was inhibited by OPs, the chromogenic reaction would be inhibited, leading to a decrease of color intensity. As a result, the color intensity was correlated with the concentration of OPs.

#### 3.2.2. Condition Optimization

For the sake of better sensitivity and efficiency, indoxyl acetate concentration and color developing time were optimized by detecting malathion-free solution, and inhibition time was optimized by detecting 1 μg/mL malathion solution. All the experiments were conducted in the detection zones of the chips, and the R values were obtained in Adobe Capture CC. As shown in Figure S3a, the R value increased with the increase of indoxyl acetate concentration from 1 mM to 8 mM, and it remained approximatively steady when indoxyl acetate concentration was above 8 mM. Therefore, 8 mM indoxyl acetate was selected for subsequent experiments. Analogously, 15 min and 20 min were chosen as the optimal inhibition time and optimal color developing time respectively (Figure S3b,c).

#### 3.2.3. Effect of the Imaging Platform

**Figure 8:**
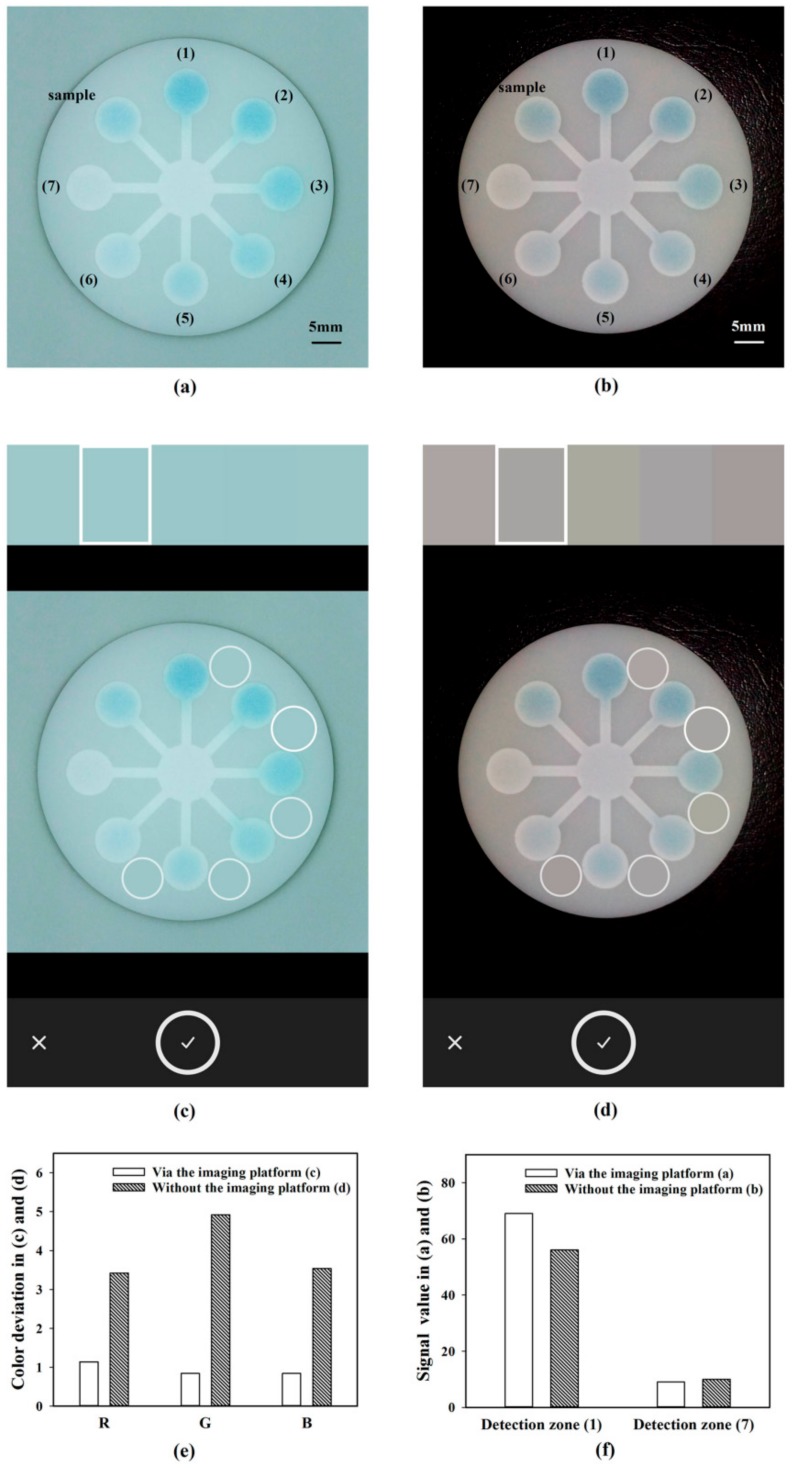
The image of the chromogenic chip captured via the imaging platform (**a**) and without the imaging platform (**b**). The uniformity of illumination in the imaging platform (**c**) and without the imaging platform (**d**) was investigated by analyzing the colors of the selected circles in Adobe Capture CC. (**e**) The color deviations of the selected circles in (**c**,**d**). (**f**) The R values of the first and seventh detection zones in (**a**,**b**). (For interpretation of the references to color in this figure legend, please refer to the web version of the article.).

After the chromogenic reaction, the image of the chip was captured via the imaging platform (Figure 8a) and without the imaging platform (Figure 8b), respectively. When the image was captured without the imaging platform, the flashlight of the smartphone was turned on. Otherwise the illumination condition would be completely different in different environment and interfered by the shadow of the smartphone. However, with the flashlight turned on, the illumination without the imaging platform was still not uniform. In order to investigate the deviation of illumination, the colors of the areas between the detection zones on the chip were analyzed in Adobe Capture CC (Figure 8c,d). As displayed on the top of user interface, the colors of the five areas in the image captured via the imaging platform were almost the same (Figure 8c), whereas those in the image captured without the imaging platform were of conspicuous difference (Figure 8d). As shown in Figure 8e, the deviation of the color values of the selected five areas were 1.14, 0.84, and 0.84 in R, G, and B channels in the image captured via the imaging platform, which could be mainly attributed to the native slight difference in the appearance of the fabricated chip. In contrast, those were 3.42, 4.92, and 3.54 in R, G, and B channels in the image captured without the imaging platform, which could be mainly attributed to the deviation of illumination. Consequently, without the imaging platform, the illumination condition was obviously non-uniform even though a single shot was used.

As shown in Figure 8f, the signal values for 4 μg/mL malathion standard solution captured by two imaging approaches were approximately equal, whereas the signal value for malathion-free solution captured via the imaging platform was obviously higher than that captured without the imaging platform. Hence, in virtue of the developed imaging platform, the variation in signals could be more sensitively detected, which was also absolutely beneficial to obtaining more accurate detection results.

#### 3.2.4. Sensing Performance

**Figure 9:**
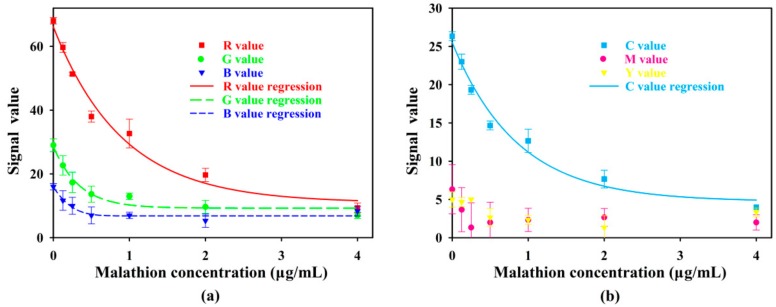
(**a**) Signal values in R, G, and B channels for different levels of malathion concentration. (**b**) Signal values in C, M, and Y channels for different levels of malathion concentration.

Signal values in R, G, B, C, M, Y, and K channels for different levels of malathion concentration were investigated (Figure 9). The signals in K channel were invariably zero, thus they were not shown in Figure 9b. The signals in R, G, B, and C channels were negatively correlated with the concentration of malathion over the range of 0–4 μg/mL, thus the regression curves in R, G, B, and C channels were established by SigmaPlot (Figure 9).

The correlation coefficient, the limit of detection (signal/noise = 3), and the recovery of the sample solution of each regression curve were calculated (see Table S1 in ESI). The regression curve in R channel had the highest correlation coefficient, the lowest limit of detection, and the most satisfactory recovery. Hence, R channel was selected for detection of malathion. The calibration equation for R value regression was calculated as:

$$y=10.9585+\frac{1413.8960}{{\left(1+e^{\frac{x+2.9489}{0.2153}}\right)}^{0.2368}},$$

where *x* represents malathion concentration (μg/mL) and *y* represents R value, and it was input into Desmos (Figure 4). The limit of detection was determined to be 51.9 ng/mL, which was far below the approved level of malathion according to US Food and Drug Administration (0.2 mM) [28] and Standardization Administration of China (0.25 mg/L) [29]. Compared with other reports for detection of malathion, the developed system showed comparable sensitivity (Table 1).

**Figure 10:**
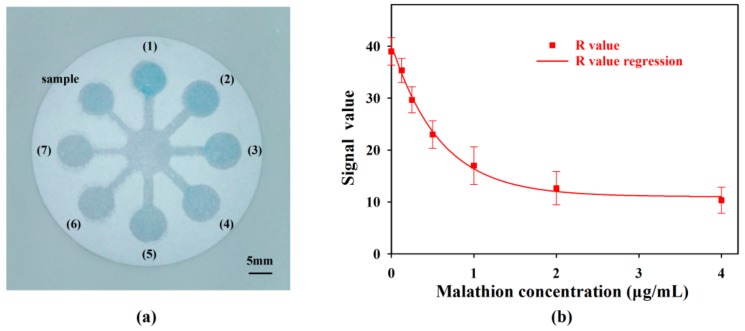
(**a**) Assay for malathion conducted on the traditional μPAD fabricated by plasma treatment. (**b**) Signal values for different levels of malathion concentration on the traditional μPAD.

The assay for malathion was also conducted on a traditional μPAD fabricated by plasma treatment, and the sensing signals were captured and processed using the same read-out. As shown in Figure 10a, the hydrophobic boundaries on the traditional μPAD were not clear enough, and the width of each straight channel was not uniform (refer to the third channel and the seventh channel in Figure 10a). The sensing signals on the traditional μPAD (Figure 10) were weaker than those on the 3D-printed chip (Figure 8a and Figure 9a). The limit of detection for malathion on the traditional μPAD was determined to be 0.226 μg/mL, which was much larger than that on the 3D-printed chip. Consequently, compared with traditional μPADs, the 3D-printed chip developed in this study was more advantageous for sensing.

In order to verify the capability of detection in real samples, four spiked domestic water samples with known malathion concentrations were tested by the developed system. As shown in Table 2, the test results showed good recovery in the range of 97.5–115.0%, with relative standard deviations of 2.56–8.70%, indicating that the developed system is reliable for on-site analysis of OPs.

### 3.3. Multi-Assay of Fruit Juice

**Figure 11:**
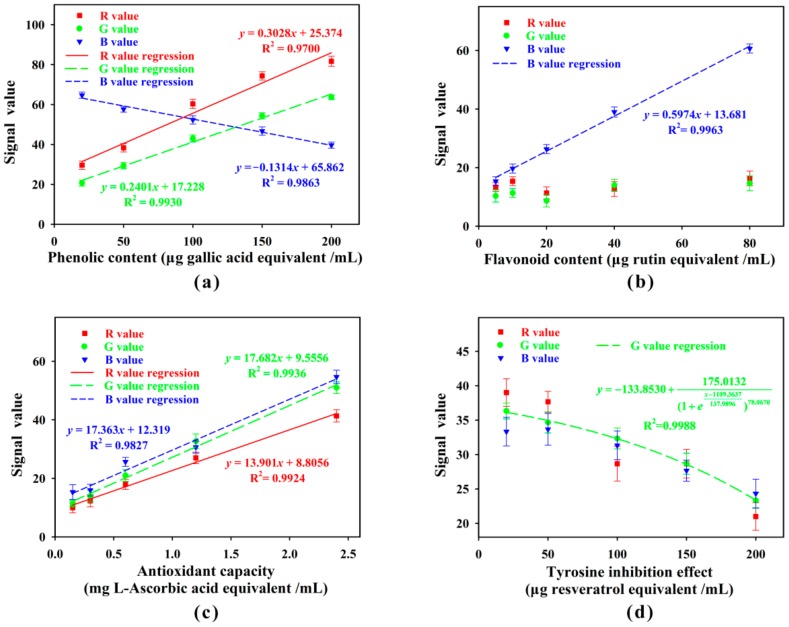
Signal values in R, G, and B channels for different levels of phenols (**a**), flavonoids (**b**), antioxidant capacity (**c**), and tyrosinase inhibition effect (**d**).

As shown in Figure 11a, signals in R, G, and B channels were all linearly dependent with phenolic content over the range of 20–200 μg gallic acid equivalent/mL, among which the regression curve in R and G channels had higher slope than that in B channel. Compared with the R value regression, although the G value regression had lower slope, it had higher correlation coefficient and lower deviation. Hence, G channel was selected for assay of phenols. As shown in Figure 11b, only the signals in B channel was correlated with flavonoid content over the range of 5–80 μg rutin equivalent/mL, thus B channel was selected for assay of flavonoids. Since the interference from R and G values was eliminated, the B value regression would be more efficient than gray value regression for assay of flavonoids. As shown in Figure 11c, signals in R, G, and B channels were all linearly dependent with antioxidant capacity over the range of 0.15–2.4 mg l-ascorbic acid equivalent/mL. Since the regression curve in G channel had the highest slope and the highest correlation coefficient, G channel was selected for assay of antioxidant capacity. As shown in Figure 11d, only the signals in G channel was negatively correlated with tyrosine inhibition effect over the range of 20–200 μg resveratrol equivalent/mL, thus G channel was selected for assay of tyrosine inhibition effect. The limit of detection (signal/noise = 3) for each detection index was shown in Table 3.

**Figure 12:**
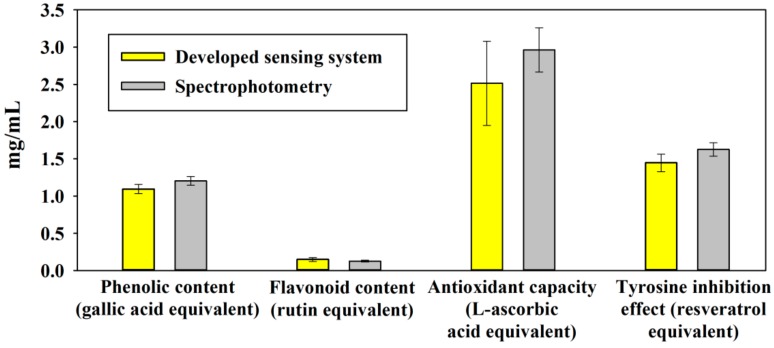
Multi-assay of *Rosa roxburghii* juice using the developed system and spectrophotometric assay.

As shown in Figure 5, all the eight detection zones in the sensing array had obvious response to the *Rosa roxburghii* juice sample. As shown in Figure 12, the detection results from the developed system well accorded with conventional spectrophotometry, with no significant difference (*p* > 0.05). Since the sensing signals for phenolic content, flavonoid content, antioxidant capacity, and tyrosine inhibition effect were simultaneously captured and processed on the smartphone, the developed system would be a portable, inexpensive, and reliable analytical tool for on-site evaluation of nutrient contents and bioactivity function of fruit juice.

## 4. Conclusions

In this study, a smartphone-based sensing system was developed, taking full advantage of the touch-screen display, high-resolution camera, high-speed computing capability, and open-source operation system of the smartphone. Since the chromogenic chip was fabricated by 3D-printing, the fabrication procedures were simplified, and the manual errors were reduced. In virtue of the imaging platform, equal condition of illumination and fixed position of the smartphone lens were well-guaranteed, thus the measurement error generated during signal capture were significantly reduced. In addition, a brand-new strategy for signal processing in downloadable apps was established. With this strategy, users could move their fingers on the smartphone touch-screen to process sensing signals and obtain detection results within a few minutes. The system was successfully applied for detection of OPs and multi-assay of fruit juice, showing excellent sensing performance. For different target analytes, the most efficient color channel could be selected for signal analysis, and the calibration equation could be directly set in user interface rather than programming environment, thus the developed system could be flexibly extended for other biochemical assays. In summary, it provides a novel methodology for smartphone-based biochemical sensing.

## Figures and Tables

**Table 1:** Comparison between this study and other reports for detection of malathion.

Technique Followed	Limit of Detection (ng/mL)	Ref.
Spectrophotometry	1780	[30]
Spectrophotometry	60	[31]
Scanner-readable plastic microchip	100	[26]
Smartphone-based sensing system	51.9	this study

**Table 2:** Prediction of malathion concentration in spiked domestic water samples.

Spiked Domestic Water Sample	Observed Concentration (μg/mL)	Expected Concentration (μg/mL)	Recovery (%)	Relative Standard Deviations (%)
I	0.23 ± 0.02	0.20	115.0	8.70
II	0.78 ± 0.02	0.80	97.5	2.56
III	1.29 ± 0.10	1.20	107.5	7.75
IV	2.55 ± 0.15	2.40	106.3	5.88

**Table 3:** Limit of detection (signal/noise = 3) for multi-assay of fruit juice.

Detection Index	Selected Signal	Limit of Detection
Phenolic content (μg gallic acid equivalent/mL)	G	11.17
Flavonoid content (μg rutin equivalent/mL)	B	3.88
Antioxidant capacity (mg L-ascorbic acid equivalent/mL)	G	0.089
Tyrosine inhibition effect (μg resveratrol equivalent/mL)	G	34.357

## Supplementary Materials

The Supplementary Materials are available online at http://www.mdpi.com/1424-8220/18/11/4002/s1, Figure S1: Microstructure of the inner wall of the imaging platform, Figure S2: The detection principle of OPs, Figure S3: Condition optimization for detection of malathion, Table S1: Comparison between four color channels for signal analysis towards malathion detection.
