# Supplementary material for: A Portable Smartphone-Based Sensing System Using a 3D-Printed Chip for On-Site Biochemical Assays
Source: Sensors (Basel). 2018 Nov 16;18(11):4002. doi: 10.3390/s18114002 (PMC6263889; doi:10.3390/s18114002)
Supplement: Supplementary file 1 [file sensors-18-04002-s001.pdf]

Electronic Supplementary Information for

A portable smartphone-based sensing system using a 3D-printed chip for on-site biochemical assays

Feiyi Wu, and Min Wang*

Institute of Microanalytical Systems, Department of Chemistry, Zhejiang University, Hangzhou 310058, China; wfy@zju.edu.cn

* Correspondence: minwang@zju.edu.cn; Tel.: +86-571-8820-6772

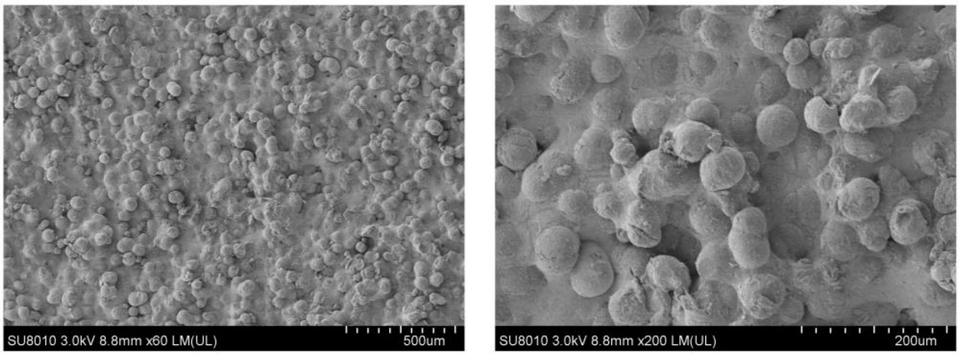

(a) (b)

Fig. S1 Microstructure of the inner wall of the imaging platform in (**a**) 60× magnification and (**b**) 200× magnification.

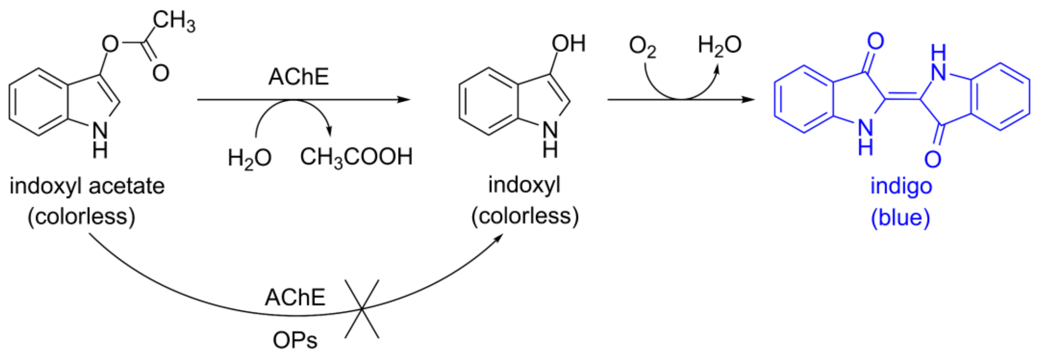

Fig. S2 The detection principle of OPs. In the presence of AChE, the colorless substrate could be converted to a blue product. Therefore, if the activity of AChE was suppressed by OPs, the chromogenic reaction would be inhibited, and the generated color intensity would descend.

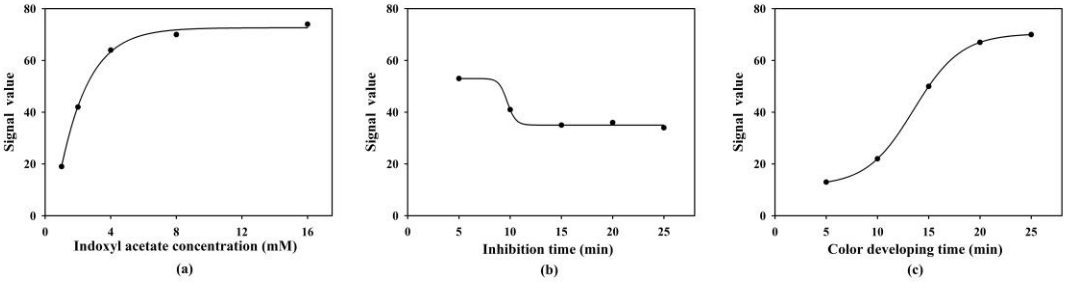

Fig. S3 Optimization of (**a**) indoxyl acetate concentration, (**b**) inhibition time, and (**c**) color developing time for detection of malathion.

Table S1 Comparison between four color channels for signal analysis towards malathion detection.

**Channel for signal analysis**	**R**	**G**	**B**	**C**
Correlation coefficient	0.9807	0.9595	0.9360	0.9789
Limit of detection (μg/mL)	0.0519	0.1260	0.0920	0.0748
Recovery (%)	94.0	51.8	40.2	87.4
